# STI571 reduces TRAIL-induced apoptosis in colon cancer cells: c-Abl activation by the death receptor leads to stress kinase-dependent cell death

**DOI:** 10.1186/1423-0127-19-35

**Published:** 2012-03-30

**Authors:** Duen-Yi Huang, Yee Chao, Ming-Hui Tai, Yang-Hao Yu, Wan-Wan Lin

**Affiliations:** 1 Department of Pharmacology, College of Medicine, National Taiwan University, Taipei, Taiwan; 2 Cancer Center, Veterans General Hospital, Taipei, Taiwan; 3 Division of Pulmonary and Critical Care Medicine, Medical Department, China Medical University Hospital, Taichung, Taiwan; 4 School of Medicine, China Medical University, Taichung, Taiwan; 5 Graduate Institute of Medical Sciences, Taipei Medical University, Taipei, Taiwan

**Keywords:** STI571, TRAIL, Antitumor, Stress kinases, Apoptosis

## Abstract

**Background:**

In an effort to achieve better cancer therapies, we elucidated the combination cancer therapy of STI571 (an inhibitor of Bcr-Abl and clinically used for chronic myelogenous leukemia) and TNF-related apoptosis-inducing ligand (TRAIL, a developing antitumor agent) in leukemia, colon, and prostate cancer cells.

**Methods:**

Colon cancer (HCT116, SW480), prostate cancer (PC3, LNCaP) and leukemia (K562) cells were treated with STI571 and TRAIL. Cell viability was determined by MTT assay and sub-G1 appearance. Protein expression and kinase phosphorylation were determined by Western blotting. c-Abl and p73 activities were inhibited by target-specific small interfering (si)RNA. In vitro kinase assay of c-Abl was conducted using CRK as a substrate.

**Results:**

We found that STI571 exerts opposite effects on the antitumor activity of TRAIL. It enhanced cytotoxicity in TRAIL-treated K562 leukemia cells and reduced TRAIL-induced apoptosis in HCT116 and SW480 colon cancer cells, while having no effect on PC3 and LNCaP cells. In colon and prostate cancer cells, TRAIL caused c-Abl cleavage to the active form via a caspase pathway. Interestingly, JNK and p38 MAPK inhibitors effectively blocked TRAIL-induced toxicity in the colon, but not in prostate cancer cells. Next, we found that STI571 could attenuate TRAIL-induced c-Abl, JNK and p38 activation in HCT116 cells. In addition, siRNA targeting knockdown of c-Abl and p73 also reduced TRAIL-induced cytotoxicity, rendering HCT116 cells less responsive to stress kinase activation, and masking the cytoprotective effect of STI571.

**Conclusions:**

All together we demonstrate a novel mediator role of p73 in activating the stress kinases p38 and JNK in the classical apoptotic pathway of TRAIL. TRAIL via caspase-dependent action can sequentially activate c-Abl, p73, and stress kinases, which contribute to apoptosis in colon cancer cells. Through the inhibition of c-Abl-mediated apoptotic p73 signaling, STI571 reduces the antitumor activity of TRAIL in colon cancer cells. Our results raise additional concerns when developing combination cancer therapy with TRAIL and STI571 in the future.

## Background

The tumor necrosis factor-related apoptosis-inducing ligand (TRAIL) is a tumor-selective, apoptosis-inducing cytokine. By binding to the death receptors DR4 and DR5, TRAIL can recruit the intracellular adaptor molecule, Fas-associated protein with death domain (FADD), to death domains present in the cytoplasmic region of these receptors and form a death-inducing signaling complex. FADD in turn can recruit and activate proximal caspase 8, which subsequently activates effector caspase 3, either by direct processing via a protease cascade or indirectly via a mitochondrial apoptotic pathway [1,2]. Apart from the caspase activation cascade, TRAIL can also activate c-Jun NH2-terminal kinase (JNK) and p38, which are thought to be important for the induction of cell apoptosis [3-5].

The recent development of target kinase inhibitors represents a breakthrough in the clinical application for several human malignancies [6]. c-Abl is a ubiquitously expressed non-receptor tyrosine kinase containing a myristoylation site, SH_2 _and SH_3 _domains, a kinase domain, DNA- and actin-binding domains, and nuclear targeting and export signals [7]. Several reports showed that c-Abl can be stimulated by physiological and pharmacological stresses, such as UV, genotoxic agents, growth factors, and TNF-α [8-10]. c-Abl is distributed in both the cytoplasm and nucleus, where it plays distinct roles [11]. Nuclear c-Abl activation in response to DNA damage, TNF-α, or FasL leads to cell growth arrest and/or apoptosis [9,12,13]. In contrast, cytoplasmic c-Abl activated by growth factors or by extracellular matrix proteins is involved in cytoskeletal remodeling and cell growth [14,15]. Even though the mechanism by which c-Abl drives cell death is not completely understood, it might involve a combination of signals. In fact, c-Abl regulates downstream molecules which are associated with cell death/survival, including p73, p63, p53, PKCδ, retinoblastoma (RB), c-Jun, IκBα and mitogen-activated protein kinases (MAPKs) [8-10,13,16]. The direct transactivation of PUMA and Bax, and the expression of death receptors by p73 were demonstrated to contribute to c-Abl-mediated apoptosis [17,18].

STI571 (imatinib, Gleevec^®^) is a specific inhibitor of tyrosine kinases, such as Bcr-Abl, c-Abl, platelet-derived growth factor receptor, and c-Kit [19]. It was approved for the treatment of Philadelphia chromosome-positive chronic myelogenous leukemia (CML) and gastrointestinal stromal tumors with constitutively active Bcr-Abl and c-Kit [20-22]. As a front-line therapy, STI571 is tremendously successful; however, STI571-resistant clones that allow the disease to progress are appearing and increasing. Thus the management of patients who are resistant to STI571 with many conventional chemotherapeutic agents still needs to be resolved. Moreover, recent studies showing that c-Abl is highly active in many aggressive breast cancer cell lines and involved in cancer cell metastasis, proliferation, and survival have raised strong interest in investigating STI571's effects in other solid tumors [15].

In an effort to achieve better cancer therapy, the possibility of combining TRAIL and STI571 in various cancer types is worth investigating. In fact, studies showed that when co-treating STI571 with TRAIL, K562 (a Bcr-Abl-positive human leukemia cell line) and melanoma cells are more sensitive to death [23,24]. Moreover, reports indicate that TRAIL can induce cell death in CML cells that are refractory to STI571, and vice versa STI571 can overcome TRAIL resistance in K562 cells [25-27]. In this report we extend to study this combination in colon and prostate cancer cells. Both STI571 and TRAIL alone have been reported to exert antitumor activity in colon cancer cells [28,29]. Intriguingly, in this study we found that STI571 can attenuate TRAIL-induced cytotoxicity in colon cancer cells, whereas it cannot affect TRAIL's effect in prostate cancer cells. We presented evidence that c-Abl mediation of TRAIL-induced JNK and p38 activation is involved in the death of colon cancer cells, but not of prostate cancer cells. Moreover, p73 is the downstream effector of c-Abl which propagates signals to JNK and p38.

## Methods

### Reagents

TRAIL was purchased from PeproTech (London, UK). STI571 was kindly provided by Norvartis Pharma AG (Basel, Switzerland). Rabbit monoclonal antibodies specific for caspase 3 and 8, phosphorylated p38, JNK, ERK, and c-Abl were obtained from Cell Signaling Technology (Beverly, MA, USA). Mouse antibodies for c-Abl, JNK1, p38, and β-actin were from Santa Cruz Biotechnology (Santa Cruz, CA, USA). The p73 antibody was purchased from BD Pharmingen Technical (San Jose, CA, USA). SB203580, SP600125, and z-Val-Ala-Asp-fluromethylketone (zVAD) were purchased from Calbiochem (San Diego, CA, USA). GST-CRK (120-225) protein was obtained from Merck Millipore (Darmstadt, Germany). All other chemicals were obtained from Sigma Aldrich (St. Louis, MO, USA).

### Cell culture

Human colon cancer HCT116 and SW480 cells, CML K562 cells, and prostate cancer PC3 and LNCaP cells obtained from American Type Culture Collection (Manassas, VA, USA) were grown in DMEM. All media were supplemented with 10% (v/v) heat inactivated FBS, 100 U/ml penicillin and 100 μg/ml streptomycin. Cells were incubated at 37°C in a humidified atmosphere of 5% CO_2 _in air and were routinely sub-cultured every 2-3 days.

### Measurement of cell viability

Cell viability was determined by 3-(4,5-dimethylthiazol-2-yl) 2,5-diphenyltetrazolium bromide (MTT) at 1 mg/ml for 30 min, then cells were dissolved in 100% DMSO. The net absorbance (OD550 nm-OD630 nm) was determined and indicated the enzymatic activity of mitochondria and cell viability.

### Apoptotic assay

After drug treatment, cells were harvested and washed twice with PBS and fixed in iced 70% ethanol, then stored at -20°C overnight. DNA extraction buffer (0.2 M Na_2_HPO_4 _and 0.1 M citric acid; pH 7.8) was added at room temperature for 30 min. Cells were then incubated in PBS containing 1 mg/ml RNaseA and 40 μg/ml propidium iodide (PI) for 30 min in the dark at room temperature. Using a FACScan flow cytometer (BD Biosciences), 10^4 ^cells were counted, and a lower DNA content than that of the G_0_/G_1 _phase indicated apoptotic cells.

### Western blotting

Cells were lysed by the addition of cold RIPA buffer [150 mM NaCl, 50 mM Tris HCL, 0.1% SDS, 1% Triton X-100, 1 mM PMSF, 2 mM NaF, Na_3_VO_4_, β-glycerophosphate and 2 mM EDTA, and fresh protease inhibitor cocktail (Catalog no. P8340, purchased by Sigma Aldrich)], and cell lysate was centrifuged at 14,000 × g at 4°C for 20 min. The supernatant was harvested and analyzed for protein content using protein assay dye. Protein was denatured in sample buffer, then separated on SDS-PAGE, and transferred to polyvinylidene difluoride membranes using a semidry trans-blot system. The blots were blocked for 1 h at room temperature with Tris-Buffered saline (TBS, 50 mM Tris-HCl, pH 7.5, 150 mM NaCl) containing 5% non-fat milk. The blots were washed three times with TBST (50 mM Tris-HCl, pH 7.5, 150 mM NaCl, and 0.02% Tween 20) and incubated with the indicated antibody at 4°C overnight. Next day, the blots were incubated for 1 h at room temperature with secondary antibody (1:5000 dilutions), and detected by ECL detection reagent. To ensure that equal amounts of sample protein were applied for electrophoresis, β-actin was used as an internal control.

### Gene silencing

The siRNA duplexes specific for human c-Abl (cat. no. L-003100-00) or p73 (cat. no. L-003331-00) were obtained from Dharmacon RNA Technologies. The siRNA for each group contained four RNA sequences in a Smart Pool selected from the NCBI RefSeq Database by a proprietary algorithm. The control non-targeting pool is a pool of four functional non-targeting siRNAs with guanine cytosine contents comparable to that of the functional siRNA but lacking specificity for known gene targets. To achieve gene silencing, we transfected cells with the indicated siRNA for 24 h followed by drug treatment; then the gene silencing effects were evaluated by Western blot analysis.

### Immunoprecipitation

For immunoprecipitation experiments, cells were washed with ice-cold PBS once and then lysed in 1 ml RIPA lysis buffer (50 mM Tris-HCl, pH 7.6, 150 mM NaCl, 1% Triton X-100, 0.1% SDS, 2 mM EDTA, 2 mM NaF, 2 mM β-glycerolphosphate, 2 mM Na_3_VO_4_, 1 mM PMSF, and protease inhibitor cocktails) and centrifuged at 10,000 rpm, 4°C for 5 min. The supernatant was collected and was pre-cleaned with 0.5 μg normal IgG and 10 μl protein A-agarose beads at 4°C for 30 min for each sample. After centrifugation, supernatant was incubated with specific antibody at 4°C overnight, and then 10 μl protein A-agarose beads were added and rocked for another 1 h. The immunocomplexes were washed two times with cold RIPA buffer containing 150 mM NaCl, two times with RIPA buffer containing 300 mM NaCl and finally RIPA buffer containing 150 mM NaCl again. SDS gel-loading buffer was added to the precipitated complexes and heat the samples at 95°C for 5 min. After spinning down the samples and loading the supernatants onto the SDS-PAGE, immunoblotting analysis was performed as described above.

### In vitro c-Abl kinase assay

To evaluate kinase activity of c-Abl, HCT116 cells were lysed in Tris-buffered saline-0.1% Triton X-100, and cell lysates were pre-cleaned at 4°C for 30 min and then immunoprecipitated with 1 μg anti-c-Abl (K-12) antibody at 4°C, 4 h. Afterwards 10 μl protein A-agarose beads were added and rocked at 4°C for another 1 h. The immunocomplexes were washed 5 times with cold lysis buffer, and then twice with the kinase reaction buffer (20 mM HEPES, pH 7.5 and 10 mM MgCl_2_). The beads were then incubated at 30°C in 40 μl kinase reaction buffer supplemented with 10 μCi of [γ-^32^P] ATP, 2 mM Na_3_VO_4_, 1 mM DTT, 10 μM ATP, protease inhibitor cocktails and 1 μg GST-CRK (120-225). The reaction was stopped by the addition of 10 μl 5× SDS-gel loading buffer and boiling for 5 min. Reaction products were run on 10% SDS-PAGE, followed by autoradiography.

### Statistical evaluation

Data were expressed as the mean ± S.E.M. of at least three experiments. Analysis of variance (ANOVA) was used to assess the statistical significance of the differences, with a *p *value of < 0.05 considered statistically significant.

## Results

### STI571 reduces TRAIL-induced cell apoptosis in colon cancer but not in prostate cancer cells

**Figure 1:**
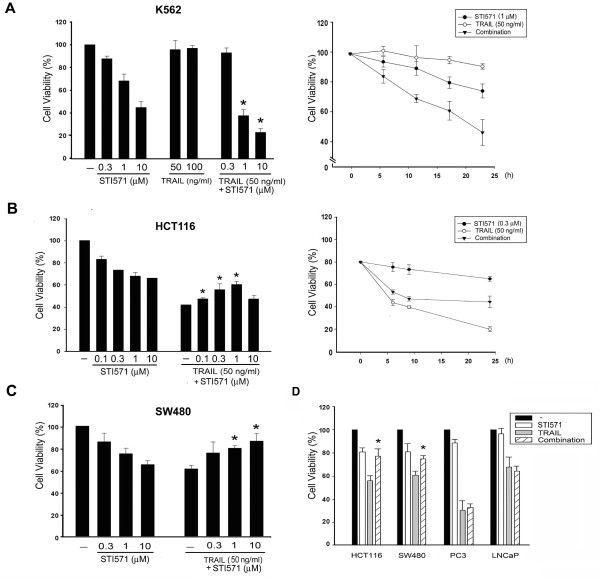
**Distinct effects of STI571 on TRAIL-induced cytotoxicity in cancer cells**. K562 cells (A), colon cancer HCT116 cells (B), and SW480 cells (C) were pretreated with STI571 for 30 min, followed by TRAIL for 24 h, and then the cell viability was assessed by an MTT assay. Cell viability was determined in some cases of K562 (A, right panel) and HCT116 cells (B, right panel) after drug treatment for different periods. In (D), various cancer cells were treated with STI571 (1 μM) and TRAIL (50 ng/ml) for 24 h, and then the cell viability was determined. * *p *< 0.05, indicating significant attenuation of TRAIL-induced cytotoxicity by STI571

A previous study revealed the beneficial cytotoxic effects of STI571 and TRAIL against K562 cells, the prototype cell model of CML [23]. Before being able to understand the combined cytotoxic effects in other cancer cell types, we first verified this action in K562 cells. Results shown in Figure 1A revealed that K562 cells were sensitive to STI571 at 1 ~ 10 μM, while they were resistant to TRAIL at concentrations up to 100 ng/ml as previously reported [27]. Co-treatment with STI571 and TRAIL led to increased cell death in concentration- and time-dependent manners. In human colon cancer HCT116 cells, STI571 (0.1 ~ 10 μM) alone induced a moderate loss of cell viability, and TRAIL induced a more prominent toxicity at 50 ng/ml. The average of cell viability under 0.3 μM STI571 and 50 ng/ml TRAIL treatment for 24 h achieved 88 ± 5% (n = 15) and 52 ± 7% (n = 20) of control, respectively. When pretreating cells with STI571 (0.1 ~ 1 μM) for 30 min, followed by TRAIL (50 ng/ml) for 24 h, we found that their respective responses in decreasing cell viability were not additive (Figure 1B, left panel). Intriguingly, STI571 attenuated TRAIL-induced cell death in a concentration-dependent manner within 0.1 ~ 1 μM, but not at 10 μM. On average, STI571 (0.3 μM) reduced TRAIL (50 ng/ml)-induced cytotoxicity by approximately 20 ~ 25%, i.e. increasing cell viability from 52 ± 7% to 72 ± 6%. This cytoprotective effect of STI571 was also time dependent (Figure 1B, right panel). STI571 also exerted a protective effect in SW480 colon cancer cells against TRAIL-induced cytotoxicity (Figure 1C). Intriguingly, unlike the protection seen in colon cancer cells, we found that TRAIL-induced cell death in prostate cancer PC3 and LNCaP cells were barely reversed by STI571, which alone had no significant effect on cell viability in both cell types (Figure 1D).

**Figure 2:**
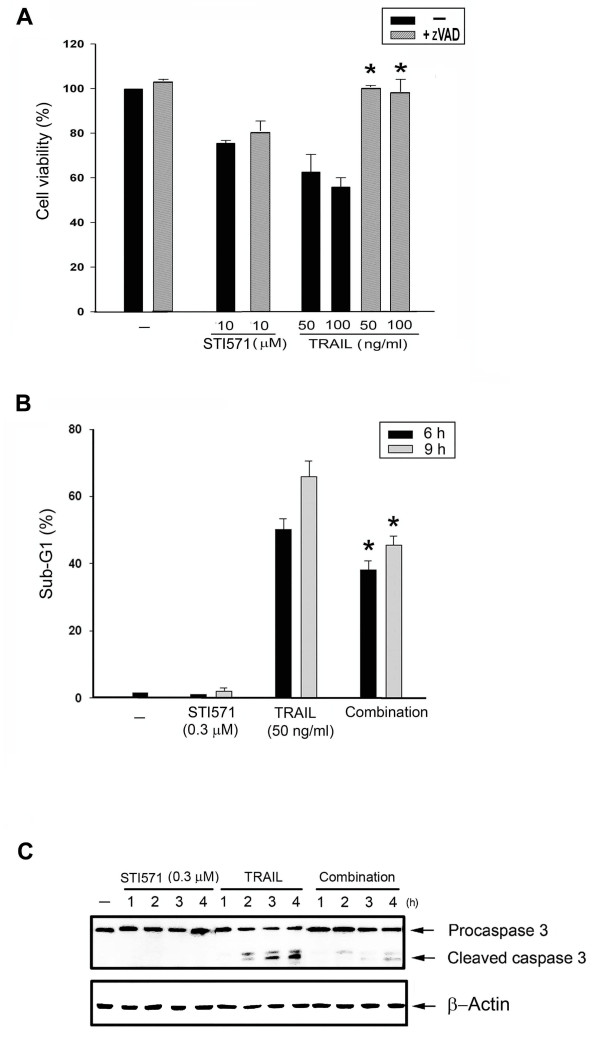
**TRAIL-induced apoptosis in HCT116 cells is attenuated by STI571**. (A) Cells were pretreated with zVAD (20 μM) for 30 min, followed by the addition of STI571 or TRAIL. After 24 h, cell viability was determined by the MTT assay. (B) After treatment with STI571 and/or TRAIL for 6 or 9 h, cells were labeled with PI, and the cell population with sub-G1 expression was determined by flow cytometry. (C) After treatment with STI571 (0.3 μM) and/or TRAIL (50 ng/ml) for 1 ~ 4 h, cell lysates were harvested for immunoblotting of caspase 3. * *p *< 0.05, indicating significant reduction of TRAIL-induced apoptosis by zVAD (A) and STI571 (B). Traces in (C) were representative of at least three separate experiments with similar results.

We used pharmacological and biochemical approaches to verify whether the reduction of TRAIL-induced cell death by STI571 involves a caspase-dependent apoptotic pathway. We found that zVAD (a pan-caspase inhibitor) completely reversed TRAIL-induced cell death, but had no effect on STI571 (Figure 2A). Moreover, with a similar effect on the MTT assay, STI571 reduced TRAIL-induced sub-G_1 _fractions (Figure 2B). We also analyzed the proteolytic processing of procaspase 3 (an effector caspase), and found that TRAIL treatment alone resulted in the processing of procaspase 3 (Figure 2C). However, when pretreated with STI571, the proteolysis of procaspase 3 was reduced.

### TRAIL activates c-Abl in colon and prostate cancer cells

**Figure 3:**
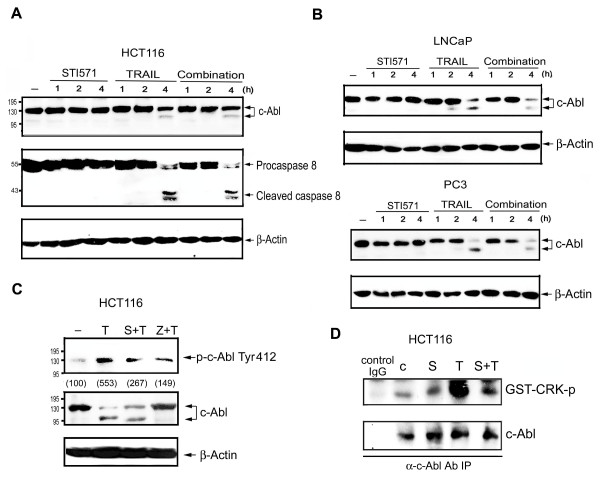
**TRAIL induces c-Abl activation and cleavage in colon and prostate cancer cells**. HCT116 (A), LNCaP, and PC3 (B) cells were treated with TRAIL (50 ng/ml) and/or STI571 (0.3 μM) for different time periods; then protein levels of c-Abl, caspase 8, and β-actin were determined. In (C), HCT116 cells were treated with STI571 (S, 0.3 μM), zVAD (Z, 20 μM), and/or TRAIL (T, 50 ng/ml) for 4 h. Then protein levels of both the total and Tyr412 phosphorylated forms of c-Abl were determined. Traces were representative of three separate experiments with similar results. The extent of Tyr412 phosphorylation as an index of kinase activation was quantified by standardizing the total non-cleaved c-Abl level and presented in parentheses. (D) After treating HCT116 cells with STI571 (S, 0.3 μM) and/or TRAIL (T, 50 ng/ml) for 3 h, c-Abl kinase activity was determined by in vitro kinase assay using GST-CRK as substrate.

To determine if TRAIL can activate c-Abl, we determined levels of c-Abl phosphorylation at Tyr412, which can stimulate kinase to full catalytic activity [30]. Moreover, we also determined if c-Abl could be cleaved by TRAIL-induced caspase activation. Previous studies showed that caspase-mediated cleavage of c-Abl produced kinase fragments for increased activity [31-33]. As shown in Figure 3A, TRAIL time-dependently induced c-Abl cleavage accompanied by caspase 8 activation in HCT116 cells. Neither action of TRAIL was affected by the presence of STI571. Similarly, TRAIL-elicited c-Abl cleavage in LNCaP and PC3 cells was not changed by STI571 (Figure 3B). Next, we tested if TRAIL could induce c-Abl activation, and if this effect was dependent on caspase. As shown in Figure 3C, c-Abl phosphorylation at Tyr412 in HCT116 cells was increased following TRAIL treatment, and this effect was inhibited by STI571 and zVAD. On the other hand, TRAIL-induced c-Abl cleavage was not changed by STI571, but was inhibited by zVAD. To determine the effects of TRAIL and STI571 on c-Abl activity, in vitro kinase activity assay using GST-CRK (120-225) as a substrate was performed. As reported, CRK adaptor protein is a kinase substrate of c-Abl, and its phosphorylation at Tyr 221 by c-Abl functions as a negative regulator of cell motility and cell survival [34,35]. We found that c-Abl activity was increased following TRAIL treatment for 3 h, and this effect was inhibited in the presence of STI571 (Figure 3D). These results suggest that the enzymatic activation of caspase is required for c-Abl cleavage and activation.

### Protection of HCT116 cells against TRAIL by STI571 is associated with JNK and p38 signaling

**Figure 4:**
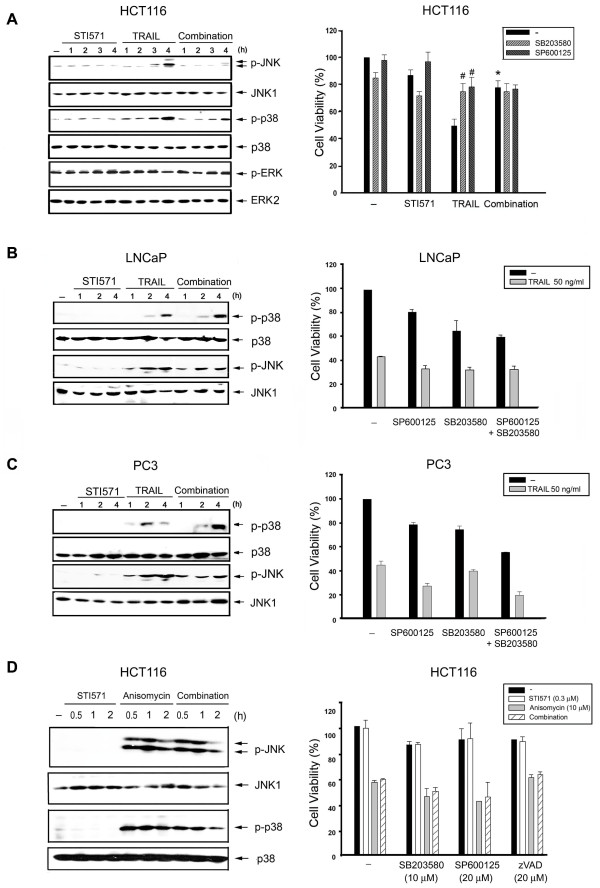
**Protection of HCT116 cells against TRAIL by STI571 is associated with JNK and p38 signaling**. HCT116 (A, D), LNCaP (B), and PC3 cells (C) were treated with TRAIL (50 ng/ml), STI571 (0.3 μM) and/or anisomycin (10 μM) for the time indicated. Then protein levels of JNK, p38, and ERK were determined (left panel). Traces were representative of three separate experiments with similar results. In some experiments, cells were pretreated with SB203580 (10 μM), SP600125 (20 μM), STI571 (0.3 μM) and/or zVAD (20 μM) as indicated for 30 min, and then TRAIL (50 ng/ml) or anisomycin (10 μM) was added. After 24 h incubation, cell viability was determined by the MTT assay (right panel). * *p *< 0.05, indicating a significant reduction in cell apoptosis by STI571. ^# ^*p *< 0.05, indicating a significant reduction in TRAIL-elicited apoptosis by kinase inhibitors.

Since JNK and p38 MAPK are important in inducing apoptosis, we investigated their involvement in TRAIL-induced cell death, and their linkage to the action of STI571. As a result, TRAIL alone significantly induced JNK and p38 phosphorylation, but did not affect ERK activation. Pretreatment with STI571 resulted in reductions in JNK and p38 activation (Figure 4A, left panel). Moreover, we found that SP600125 (JNK inhibitor) and SB203580 (p38 inhibitor) could partially reverse TRAIL-induced cell death, but did not produce further increased protection in combination with STI571 (Figure 4A, right panel). Nevertheless, in LNCaP and PC3 cells, neither SB203580 nor SP600125 treatment, either alone or in combination, altered TRAIL-induced cytotoxicity (Figure 4B, C, right panel). And unlike the effects in HCT116 cells, STI571 cannot alter TRAIL-induced p38 and JNK activation (Figure 4B, C, left panel). These results suggest the involvement of JNK and p38 in TRAIL-induced cell death in colon cancer cells, and the protective mechanism of STI571 might be related to both kinases.

Following observing the ability of STI571 to inhibit TRAIL-activated stress kinases in HCT116 cells, we were wondering the stimuli-specific action of STI571. Thus we tested effects of STI571 on stress kinase activation caused by anisomycin, which is known to be a potent inducer of JNK and p38 [36]. Results revealed that anisomycin rapidly activated JNK and p38 phosphorylation in HCT116 cells, and the extents of activation were not affected by STI571 (Figure 4D, left panel). Moreover, anisomycin alone induced cell death, but this effect was not reversed by pretreatment with STI571, SB203580, or SP600125 (Figure 4D, right panel). These results suggest that STI571-elicited attenuation of stress kinase activation is not a general action, but is specific in colon cancer cells in response to the extrinsic death inducer, TRAIL.

### Reduced cell susceptibility to TRAIL by STI571 is dependent on c-Abl and p73

**Figure 5:**
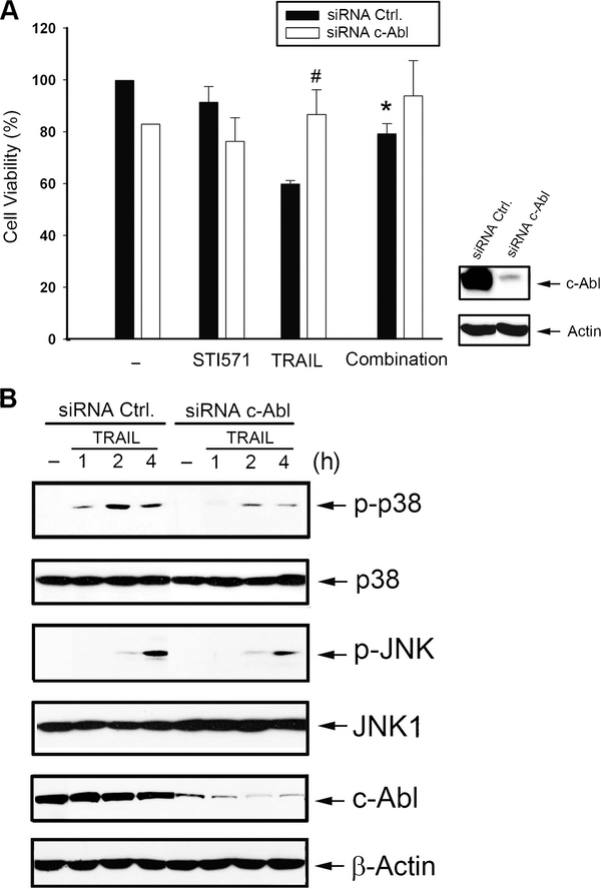
**STI571-induced reduction of cell susceptibility to TRAIL is dependent on c-Abl**. (A) HCT116 cells were transfected with c-Abl siRNA, and 24 h after transfection the intracellular c-Abl protein level was determined. In some experiments, cells were treated with TRAIL (50 ng/ml) and/or STI571 (0.3 μM) as indicated for another 24 h, and cell viability was determined. * *p *< 0.05, indicating a significant reduction in apoptosis by STI571. ^# ^*p *< 0.05, indicating a significant reduction of TRAIL-elicited apoptosis by c-Abl siRNA. (B) In some experiments under c-Abl siRNA treatment, JNK and p38 activation by TRAIL were determined. Data of immunoblotting were representative of three separate experiments with similar results.

To understand the role of c-Abl in STI571's action, we used RNA-silencing technology. Results showed that TRAIL-induced cytotoxicity was reversed by c-Abl siRNA (Figure 5A), and under this condition, STI571-induced protection was no longer observed. Moreover, c-Abl siRNA reduced p38 and JNK activations after TRAIL treatment compared to cells transfected with scrambled control siRNA (Figure 5B). These data suggest that c-Abl is required for HCT116 cells to be responsive to TRAIL-induced p38 and JNK signaling, and both in turn contribute to cell death.

**Figure 6:**
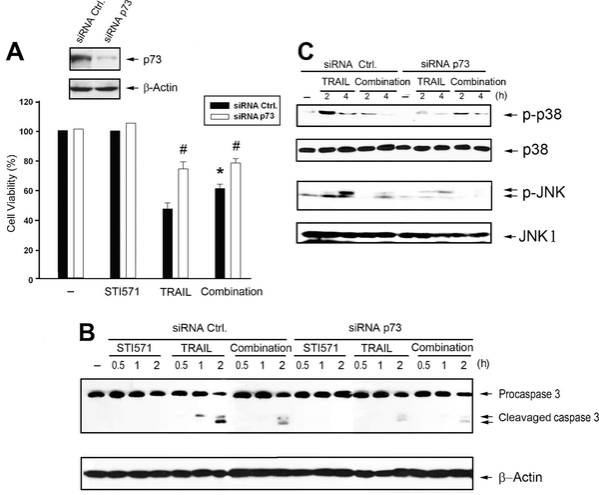
**STI571-induced cell resistance to TRAIL is dependent on p73**. (A) HCT116 cells were transfected with p73 siRNA, and were treated with TRAIL (50 ng/ml) and/or STI571 (0.3 μM) as indicated. Cell viability at 24 h and protein expression of p73 were determined. * *p *< 0.05, indicating a significant reduction of TRAIL-induced apoptosis by STI571. ^# ^*p *< 0.05, indicating a significant difference of TRAIL-elicited cell death between p73 siRNA and scrambled control cells. In some experiments, caspase 3 (B), JNK and p38 activations (C) were measured by assessing their cleavage form and phosphorylation state. Data of immunoblotting were representative of three separate experiments with similar results.

A recent study reported that p73, a downstream target of c-Abl, plays a role in regulating cell death [37]. To understand the roles played by p73 in TRAIL-induced cell death and STI571-induced TRAIL resistance, we transfected p73 siRNA in HCT116 cells. Results showed that under p73 knockdown condition, TRAIL-induced cell death (Figure 6A), caspase 3 cleavage (Figure 6B), JNK and p38 activation (Figure 6C) were inhibited as seen with STI571. Meanwhile with p73 silencing, the inhibitory effects of STI571 on cell death, and activation of MAPKs and caspase 3 were not further observed. The fact that p73 targeted by siRNA induced similar inhibitory effects as did STI571 on TRAIL responses suggests that p73 is crucial for TRAIL-elicited cell death and mediates the actions of STI571.

## Discussion

TRAIL is a potential anticancer agent, and drug combination therapy to improve its effectiveness has recently garnered much attention. In this respect, its advantaged combination with STI571 has been shown in CML and melanoma. TRAIL and STI571 can mutually overcome respective death resistance in CML [23,25,27]. Co-treatment with STI571 also enhances the susceptibility of melanoma cells to TRAIL [24]. Based on previous promising results of this combination effect, we were interested to address whether other types of cancers also confer higher susceptibility towards co-treatment of both antitumor agents. To this end, in this study we chose colon cancer and prostate cancer cells, where STI571 and TRAIL alone have been demonstrated to exert antitumor activity [28,29,38,39].

Here we found that the action of TRAIL in colon cancer cells is sensitive to zVAD, confirming the process of apoptosis. However, a slight reduction in cell viability by STI571 (of around 12% at 0.3 μM) was not affected by zVAD, ruling out the process of apoptosis. Instead, a cell proliferation analysis indicated that STI571 can inhibit HCT116 cell growth (data not shown) as reported in HT29 colon cancer cells [40]. When treating HCT116 cells with STI571 and TRAIL, an antagonistic result was obtained, suggesting that STI571 can regulate the death effect of TRAIL. Such antagonistic effect of STI571 exhibited the concentration-dependency at 0.1 ~ 1 μM. However, a higher concentration of STI571 (10 μM) did not display this effect. Currently we cannot explain the latter observation for the interaction of STI571 and TRAIL, but it is suggested that multiple mechanisms participate in regulating TRAIL's effect by STI571.

Many cytotoxic chemotherapeutic drugs sensitize cancer cells to TRAIL by increasing its receptor expression [29]. In this respect, STI571 did not change caspase 8 activation caused by TRAIL, ruling out STI571's action is related to death receptor expression or activation of upstream death signals. Moreover, we conducted immunoblotting with DR4 and DR5 antibodies and flow cytometry to detect surface DR5 expression. The lack of any effects in these experiments (data not shown) indicates that STI571 does not change expression of the death receptors.

TRAIL-induced apoptosis has been shown to involve p38 and JNK followed by caspase 3 activation in HeLa and HCT116 cells [4,29]. Thus, sensitizing cancer cells to TRAIL through activating JNK and p38, which subsequently regulate pro-apoptotic and anti-apoptotic Bcl-2 family members and p53, becomes a promising approach to cancer therapy [41-44]. Using pharmacological inhibitors, we showed the involvement of JNK and p38 in TRAIL-induced cytotoxicity and in STI571-induced cell protection in HCT116 cells. Under conditions of p38 or JNK inhibition, TRAIL-elicited cell death was inhibited. Moreover, STI571 also inhibited activation of both stress kinases induced by TRAIL, but no longer exerted its cytoprotection when TRAIL-elicited MAPK activation was already abolished. We thus suggest that inhibition of JNK and p38 are involved in STI571-induced protection.

Activation of c-Abl by certain DNA-damaging agents contributes to cell apoptosis via p53-dependent and -independent mechanisms. First of all, Yuan and colleagues found that c-Abl is activated by infrared and in turn leads to G_1 _growth arrest via a p53-dependent mechanism. However, they also noted that transfecting p53-/- cells with wild-type c-Abl could still sensitize cell apoptosis in response to DNA damage, whereas expressing the kinase dead c-Abl could not [10]. Later, they identified p73, a homologue of p53, as a downstream mediator of c-Abl for inducing cell apoptosis [37,45]. c-Abl was shown to stabilize p73 through phosphorylation-dependent posttranslational regulation [17,18,45-47]. To determine if c-Abl and p73 are targets of STI571 in initiating cytoprotection, we silenced c-Abl and p73 using the siRNA approach. As the results seen in experiments using the kinase inhibitor, we found that downregulation of c-Abl or p73 rendered cells less sensitive to TRAIL for JNK and p38 activation as well as for cell apoptosis. We therefore conclude that c-Abl-dependent p73 activation is involved in TRAIL-induced apoptosis in HCT116 cells. Moreover, in agreement with previous findings [29,48], we did not observe effects of TRAIL to increase protein expression of p53 and Bax in p53-proficient HCT116 cells (data not shown).

The major function identified for p73 is induction of apoptosis [19,20,49,50]. Studies demonstrated the cross-talk between p73 and stress kinases (JNK and p38), leading to the upregulation of apoptotic Bcl-2 proteins and cell death. JNK can form a complex with p73 and phosphorylate p73 at multiple residues [51]. Activation of c-Abl by DNA damage was also reported to activate p38, and p38 is then sufficient to induce p73 phosphorylation and enhance its transcriptional activity [49,52,53]. Thus, activation of p73 by c-Abl may play an important role in cancer therapy, especially in cancer cells that lose p53 function, but express p73. In this study, our results indicate that a c-Abl-dependent p73 pathway is involved in JNK and p38 activation, and mediates the death mechanism of TRAIL in colon cancer cells. In this respect, activated p73 via caspase pathway has been shown to localize to mitochondria and augment cytochrome c release and cell death [50]. Therefore, in addition to being a transcription factor, p73 is speculated to have novel protein-protein interacting roles which contribute to enhancement of cell apoptosis. Although JNK can directly interact with p73 [51], it still needs to identify the interactive proteins linking p73 to p38. Apart from the involvement of c-Abl-p73 in stress kinase activation caused by TRAIL, we still cannot rule out other signaling pathways that link death receptors to JNK and p38. In this respect, TRAIL might also activate JNK through the adaptor molecules, TNF receptor-associated death domain (TRADD), FADD, TNF receptor-associated factor 2 (TRAF2) and receptor-interacting protein (RIP) [54-56]. Moreover, mitogen-activated protein kinase kinase 1 (MEKK1) and MEKK4 activated by caspase 8 were demonstrated to be responsible for TRAIL-induced JNK or p38 activation [57].

In this study, we also demonstrated caspase-dependent c-Abl cleavage and activation in TRAIL-treated colon and prostate cancer cell lines. Many studies demonstrated that the phosphorylation of c-Abl at Tyr412 by receiving signals through Src kinases, receptor tyrosine kinases or autophosphorylation, is an index for full c-Abl activation [30]. Moreover, besides phosphorylation-mediated activation, c-Abl can be cleaved by caspase in the C-terminal region [31-33]. Such cleavage occurs mainly in the cytoplasmic compartment and generates a 120-kDa fragment that can lead to increased kinase activity and/or accumulation in the nucleus [31,32]. Our present results clearly demonstrate the occurrence of both phosphorylation activation and proteolytic activation of c-Abl following TRAIL stimulation in HCT116 cells. Moreover, both activating mechanisms are mediated by a caspase pathway, and the increase of Tyr412 phosphorylation is occurred on residual non-cleaved c-Abl. Notably STI571 did not alter the c-Abl cleavage caused by TRAIL, but partially reduced the extent of Tyr412 phosphorylation. These results suggest the existence of c-Abl autophosphorylation at Tyr412 in TRAIL-stimulated cells, and also imply a cleavage-independent, but caspase-mediated mechanism for c-Abl activation. In this respect, a previous report showed that TNF-α can activate c-Abl and upregulate apoptotic p73 function via a caspase-dependent elimination of retinoblastoma protein, and thus unleashing the nuclear apoptotic effector, c-Abl [8]. Currently the molecular events linking caspase to non-cleaved c-Abl activation following TRAIL stimulation remains unknown, and further investigation is required.

In contrast to reduced TRAIL sensitivity in colon cancer cells, STI571 did not change the susceptibility of PC3 and LNCaP cells to TRAIL. We ruled out such cell type-specific effects of STI571 being related to c-Abl protein expression. Similar expression levels of c-Abl were observed in HCT116, SW480, PC3, and LNCaP cells (data not shown). Instead, we suggest that the antitumor activity of TRAIL in colon and prostate cancers might involve distinctive regulation and complex apoptotic pathways. In prostate cancers, neither p38 nor JNK activation by TRAIL is involved in cell death, while STI571 can still slightly inhibit TRAIL-induced JNK activation in prostate cancers. Moreover, TRAIL-mediated c-Abl cleavage displayed the same pattern in HCT116 and LNCaP cells. Therefore, these results further support the notion that the cell type-specific effect of STI571 on antitumor activity of TRAIL is dependent on the roles of p38 and JNK in cell death per se.

## Conclusions

**Figure 7:**
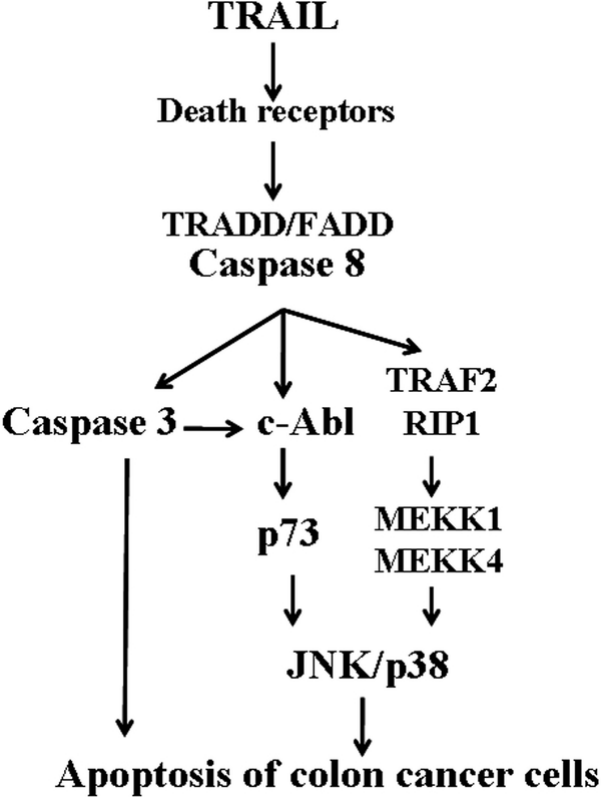
**Signaling pathway for the cytoprotective effect of STI571 in TRAIL-treated colon cancer cells**. In addition to induce classical apoptotic cascade elicited by caspases, TRAIL-induced apoptosis in colon cancer cells requires p38 and JNK activation. We propose both kinase activation by TRAIL relies on activation of c-Abl, and the downstream targeting molecule p73. Abrogating c-Abl activity by STI571 can reduce TRAIL-mediated stress kinase activation and reduce cell apoptosis. Other signaling cascades and molecules involved in the TRAIL-induced stress kinases activation, such as TRAF2, RIP1, and MEKK1/MEKK4, cannot be excluded.

We demonstrate a novel mediator role of p73 in activating the stress kinases, p38 and JNK, in the apoptotic pathway of TRAIL (Figure 7). This action is initiated by caspase-dependent c-Abl activation, and is a key mechanism contributing to death receptor-mediated cell apoptosis in colon cancer, but not prostate cancer cells. Through inhibition of the c-Abl-mediated apoptotic p73 signaling, STI571 reduces the antitumor activity of TRAIL. In this sense, this study is not in favor of the cocktail therapy of STI571 and TRAIL in human colon cancers, and also highlights the cancer-specific effect of stress kinases on the antitumor activities of TRAIL.

## Abbreviations

CML: Chronic myelogenous leukemia; DR: Death receptor; FADD: Fas-associated protein with death domain; FasL: Fas ligand; JNK: c-Jun NH2-terminal kinase; MAPK: Mitogen-activated protein kinase; MEKK: Mitogen-activated protein kinase kinase; MTT: 3-(4,5-dimethylthiazol-2-yl) 2,5-diphenyltetrazolium bromide; PI: Propidium iodide; RB: Retinoblastoma; RIP: Receptor-interacting protein; TNF-α: Tumor necrotic factor-α; TRAF2: TNF receptor-associated factor 2; TRAIL: TNF-related apoptosis-inducing ligand; zVAD: z-Val-Ala-Asp-fluromethylketone.

## Competing interests

The authors declare that they have no competing interests.

## Authors' contributions

WWL and YC designed research; DYH, MHT and YHY performed experiments; DYH and MHT analyzed data; WWL wrote the paper. All authors read and approved the final manuscript.

## References

[B1] Ashkenazi A Dixit VM Apoptosis control by death and decoy receptors Curr Opin Cell Biol 1999 11 255 260 10.1016/S0955-0674(99)80034-9 10209153

[B2] Ozoren N El-Deiry WS Defining characteristics of Types I and II apoptotic cells in response to TRAIL Neoplasia 2002 4 551 557 10.1038/sj.neo.7900270 12407450 PMC1503670

[B3] Wajant H Gerspach J Pfizenmaier K Tumor therapeutics by design: targeting and activation of death receptors Cytokine Growth Factor Rev 2005 16 55 76 10.1016/j.cytogfr.2004.12.001 15733832

[B4] Lee MW Park SC Yang YG Yim SO Chae HS Bach JH Lee HJ Kim KY Lee WB Kim SS The involvement of reactive oxygen species (ROS) and p38 mitogen- activated protein (MAP) kinase in TRAIL/Apo2L-induced apoptosis FEBS Lett 2002 512 313 318 10.1016/S0014-5793(02)02225-1 11852102

[B5] Falschlehner C Emmerich CH Gerlach B Walczak H TRAIL signalling: decisions between life and death Int J Biochem Cell Biol 2007 39 1462 1475 10.1016/j.biocel.2007.02.007 17403612

[B6] Singer CF Hudelist G Lamm W Mueller R Czerwenka K Kubista E Expression of tyrosine kinases in human malignancies as potential targets for kinase-specific inhibitors Endocr Relat Cancer 2004 11 861 869 10.1677/erc.1.00801 15613459

[B7] Pendergast AM The Abl family kinases: mechanisms of regulation and signaling Adv Cancer Res 2002 85 51 100 12374288 10.1016/s0065-230x(02)85003-5

[B8] Chau BN Chen TT Wan YY DeGregori J Wang JY Tumor necrosis factor alpha-induced apoptosis requires p73 and c-ABL activation downstream of RB degradation Mol Cell Biol 2004 24 4438 4447 10.1128/MCB.24.10.4438-4447.2004 15121862 PMC400462

[B9] Wang JY Regulation of cell death by the Abl tyrosine kinase Oncogene 2000 19 5643 5650 10.1038/sj.onc.1203878 11114745

[B10] Yuan ZM Huang Y Ishiko T Kharbanda S Weichselbaum R Kufe D Regulation of DNA damage-induced apoptosis by the c-Abl tyrosine kinase Proc Natl Acad Sci USA 1997 94 1437 1440 10.1073/pnas.94.4.1437 9037071 PMC19809

[B11] Van Etten RA Cycling, stressed-out and nervous: cellular functions of c-Abl Trends Cell Biol 1999 9 179 186 10.1016/S0962-8924(99)01549-4 10322452

[B12] Dan S Naito M Seimiya H Kizaki A Mashima T Tsuruo T Activation of c-Abl tyrosine kinase requires caspase activation and is not involved in JNK/SAPK activation during apoptosis of human monocytic leukemia U937 cells Oncogene 1999 18 1277 1283 10.1038/sj.onc.1202423 10022809

[B13] Gong JG Costanzo A Yang HQ Melino G Kaelin WG Jr Levrero M Wang JY The tyrosine kinase c-Abl regulates p73 in apoptotic response to cisplatin-induced DNA damage Nature 1999 399 806 809 10.1038/21690 10391249

[B14] Lewis JM Baskaran R Taagepera S Schwartz MA Wang JY Integrin regulation of c-Abl tyrosine kinase activity and cytoplasmic-nuclear transport Proc Natl Acad Sci USA 1996 93 15174 15179 10.1073/pnas.93.26.15174 8986783 PMC26376

[B15] Srinivasan D Sims JT Plattner R Aggressive breast cancer cells are dependent on activated Abl kinases for proliferation, anchorage-independent growth and survival Oncogene 2008 27 1095 1105 10.1038/sj.onc.1210714 17700528

[B16] Kawai H Nie L Yuan ZM Inactivation of NF-kappaB-dependent cell survival, a novel mechanism for the proapoptotic function of c-Abl Mol Cell Biol 2002 22 6079 6088 10.1128/MCB.22.17.6079-6088.2002 12167702 PMC134000

[B17] Melino G Bernassola F Ranalli M Yee K Zong WX Corazzari M Knight RA Green DR Thompson C Vousden KH p73 induces apoptosis via PUMA transactivation and Bax mitochondrial translocation J Biol Chem 2004 279 8076 8083 14634023 10.1074/jbc.M307469200

[B18] Ramadan S Terrinoni A Catani MV Sayan AE Knight RA Mueller M Krammer PH Melino G Candi E p73 induces apoptosis by different mechanisms Biochem Biophys Res Commun 2005 331 713 717 10.1016/j.bbrc.2005.03.156 15865927

[B19] Heinrich MC Griffith DJ Druker BJ Wait CL Ott KA Zigler AJ Inhibition of c-kit receptor tyrosine kinase activity by STI 571, a selective tyrosine kinase inhibitor Blood 2000 96 925 932 10910906

[B20] Demetri GD von Mehren M Blanke CD Van den Abbeele AD Eisenberg B Roberts PJ Heinrich MC Tuveson DA Singer S Janicek M Fletcher JA Silverman SG Silberman SL Capdeville R Kiese B Peng B Dimitrijevic S Druker BJ Corless C Fletcher CD Joensuu H Efficacy and safety of imatinib mesylate in advanced gastrointestinal stromal tumors N Engl J Med 2002 347 472 480 10.1056/NEJMoa020461 12181401

[B21] Goldman JM Melo JV Targeting the BCR-ABL tyrosine kinase in chronic myeloid leukemia N Engl J Med 2001 344 1084 1086 10.1056/NEJM200104053441409 11287980

[B22] Joensuu H Roberts PJ Sarlomo-Rikala M Andersson LC Tervahartiala P Tuveson D Silberman S Capdeville R Dimitrijevic S Druker B Demetri GD Effect of the tyrosine kinase inhibitor STI571 in a patient with a metastatic gastrointestinal stromal tumor N Engl J Med 2001 344 1052 1056 10.1056/NEJM200104053441404 11287975

[B23] Nimmanapalli R Porosnicu M Nguyen D Worthington E O'Bryan E Perkins C Bhalla K Cotreatment with STI-571 enhances tumor necrosis factor alpha-related apoptosis-inducing ligand (TRAIL or apo-2 L)-induced apoptosis of Bcr-Abl-positive human acute leukemia cells Clin Cancer Res 2001 7 350 357 11234890

[B24] Hamaï A Richon C Meslin F Faure F Kauffmann A Lecluse Y Jalil A Larue L Avril MF Chouaib S Mehrpour M Imatinib enhances human melanoma cell susceptibility to TRAIL-induced cell death: Relationship to Bcl-2 family and caspase activation Oncogene 2006 25 7618 7634 10.1038/sj.onc.1209738 16983347

[B25] Kikuchi S Nagai T Kunitama M Kirito K Ozawa K Komatsu N Active FKHRL1 overcomes imatinib resistance in chronic myelogenous leukemia-derived cell lines via the production of tumor necrosis factor-related apoptosis-inducing ligand Cancer Sci 2007 98 1949 1958 10.1111/j.1349-7006.2007.00623.x 17900262 PMC11158645

[B26] Park SJ Kim MJ Kim HB Sohn HY Bae JH Kang CD Kim SH Cotreatment with apicidin overcomes TRAIL resistance via inhibition of Bcr-Abl signaling pathway in K562 leukemia cells Exp Cell Res 2009 315 1809 1818 10.1016/j.yexcr.2009.02.024 19268463

[B27] Uno K Inukai T Kayagaki N Goi K Sato H Nemoto A Takahashi K Kagami K Yamaguchi N Yagita H Okumura K Koyama-Okazaki T Suzuki T Sugita K Nakazawa S TNF-related apoptosis-inducing ligand (TRAIL) frequently induces apoptosis in Philadelphia chromosome-positive leukemia cells Blood 2003 101 3658 3667 10.1182/blood-2002-06-1770 12506034

[B28] Attoub S Rivat C Rodrigues S Van Bocxlaer S Bedin M Bruyneel E Louvet C Kornprobst M André T Mareel M Mester J Gespach C The c-kit tyrosine kinase inhibitor STI571 for colorectal cancer therapy Cancer Res 2002 62 4879 4883 12208734

[B29] Su RY Chao Y Chen TY Huang DY Lin WW 5-Aminoimidazole-4-carboxamide riboside sensitizes TRAIL- and TNFα-induced cytotoxicity in colon cancer cells through AMP-activated protein kinase signaling Mol Cancer Ther 2007 6 1562 1571 10.1158/1535-7163.MCT-06-0800 17513605

[B30] Brasher BB Van Etten RA c-Abl has high intrinsic tyrosine kinase activity that is stimulated by mutation of the Src homology 3 domain and by autophosphorylation at two distinct regulatory tyrosines J Biol Chem 2000 275 35631 35637 10.1074/jbc.M005401200 10964922

[B31] Barila D Rufini A Condo I Ventura N Dorey K Superti-Furga G Testi R Caspase-dependent cleavage of c-Abl contributes to apoptosis Mol Cell Biol 2003 23 2790 2799 10.1128/MCB.23.8.2790-2799.2003 12665579 PMC152541

[B32] Machuy N Rajalingam K Rudel T Requirement of caspase-mediated cleavage of c-Abl during stress-induced apoptosis Cell Death Differ 2004 11 290 300 10.1038/sj.cdd.4401336 14657961

[B33] Podar K Raab MS Tonon G Sattler M Barilà D Zhang J Tai YT Yasui H Raje N DePinho RA Hideshima T Chauhan D Anderson KC Up-regulation of c-Jun inhibits proliferation and induces apoptosis via caspase-triggered c-Abl cleavage in human multiple myeloma Cancer Res 2007 67 1680 1688 10.1158/0008-5472.CAN-06-1863 17308109

[B34] Cipres A Abassi YA Vuori K Abl functions as a negative regulator of Met-induced cell motility via phosphorylation of the adapter protein CrkII Cell Signal 2007 19 1662 1670 10.1016/j.cellsig.2007.02.011 17399949

[B35] Kain KH Gooch S Klemke RL Cytoplasmic c-Abl provides a molecular 'Rheostat' controlling carcinoma cell survival and invasion Oncogene 2003 22 6071 6080 10.1038/sj.onc.1206930 12955086

[B36] Chung KC Kim SM Rhang S Lau LF Gomes I Ahn YS Expression of immediate early gene pip92 during anisomycin-induced cell death is mediated by the JNK- and p38-dependent activation of Elk1 Eur J Biochem 2000 267 4676 4684 10.1046/j.1432-1327.2000.01517.x 10903500

[B37] Yuan ZM Shioya H Ishiko T Sun X Gu J Huang YY Lu H Kharbanda S Weichselbaum R Kufe D p73 is regulated by tyrosine kinase c-Abl in the apoptotic response to DNA damage Nature 1999 399 814 817 10.1038/21704 10391251

[B38] Pinto AC Moreira JN Simões S Liposomal imatinib-mitoxantrone combination: formulation development and therapeutic evaluation in an animal model of prostate cancer Prostate 2011 71 81 90 10.1002/pros.21224 20607721

[B39] Voelkel-Johnson C TRAIL-mediated signaling in prostate, bladder and renal cancer Nat Rev Urol 2011 8 417 427 10.1038/nrurol.2011.81 21670755

[B40] Stahtea XN Roussidis AE Kanakis I Tzanakakis GN Chalkiadakis G Mavroudis D Kletsas D Karamanos NK Imatinib inhibits colorectal cancer cell growth and suppresses stromal-induced growth stimulation, MT1-MMP expression and pro-MMP2 activation Int J Cancer 2007 121 2808 2814 10.1002/ijc.23029 17721919

[B41] El Fajoui Z Toscano F Jacquemin G Abello J Scoazec JY Micheau O Saurin JC Oxaliplatin sensitizes human colon cancer cells to TRAIL through JNK-dependent phosphorylation of Bcl-xL Gastroenterology 2011 141 663 673 10.1053/j.gastro.2011.04.055 21683075

[B42] Badmann A Keough A Kaufmann T Bouillet P Brunner T Corazza N Role of TRAIL and the pro-apoptotic Bcl-2 homolog Bim in acetaminophen-induced liver damage Cell Death Dis 2011 2 e171 10.1038/cddis.2011.55 21654829 PMC3168997

[B43] Lamy V Bousserouel S Gossé F Minker C Lobstein A Raul F Lupulone triggers p38 MAPK-controlled activation of p53 and of the TRAIL receptor apoptotic pathway in human colon cancer-derived metastatic cells Oncol Rep 2011 26 109 114 21519792 10.3892/or.2011.1273

[B44] Sánchez-Pérez T Ortiz-Ferrón G López-Rivas A Mitotic arrest and JNK-induced proteasomal degradation of FLIP and Mcl-1 are key events in the sensitization of breast tumor cells to TRAIL by antimicrotubule agents Cell Death Differ 2010 17 883 894 10.1038/cdd.2009.176 19942932

[B45] Tsai KK Yuan ZM c-Abl stabilizes p73 by a phosphorylation-augmented interaction Cancer Res 2003 63 3418 3424 12810679

[B46] Jost CA Marin MC Kaelin WG Jr p73 is a simian [correction of human] p53-related protein that can induce apoptosis Nature 1997 389 191 194 10.1038/38298 9296498

[B47] Kaghad M Bonnet H Yang A Creancier L Biscan JC Valent A Minty A Chalon P Lelias JM Dumont X Ferrara P McKeon F Caput D Monoallelically expressed gene related to p53 at 1p36, a region frequently deleted in neuroblastoma and other human cancers Cell 1997 90 809 819 10.1016/S0092-8674(00)80540-1 9288759

[B48] Lee DH Rhee JG Lee YJ Reactive oxygen species up-regulate p53 and Puma; a possible mechanism for apoptosis during combined treatment with TRAIL and wogonin Br J Pharmacol 2009 157 1189 1202 10.1111/j.1476-5381.2009.00245.x 19438509 PMC2743838

[B49] Sanchez-Prieto R Sanchez-Arevalo VJ Servitja JM Gutkind JS Regulation of p73 by c-Abl through the p38 MAP kinase pathway Oncogene 2002 21 974 979 10.1038/sj.onc.1205134 11840343

[B50] Sayan AE Sayan BS Gogvadze V Dinsdale D Nyman U Hansen TM Zhivotovsky B Cohen GM Knight RA Melino G p73 and caspase-cleaved p73 fragments localize to mitochondria and augment TRAIL-induced apoptosis Oncogene 2008 27 4363 4372 10.1038/onc.2008.64 18362891

[B51] Jones EV Dickman MJ Whitmarsh AJ Regulation of p73-mediated apoptosis by c-Jun N-terminal kinase Biochem J 2007 405 617 623 10.1042/BJ20061778 17521288 PMC2267308

[B52] Cong F Goff SP c-Abl-induced apoptosis, but not cell cycle arrest, requires mitogen-activated protein kinase kinase 6 activation Proc Natl Acad Sci USA 1999 96 13819 13824 10.1073/pnas.96.24.13819 10570156 PMC24148

[B53] Galan-Moya EM Hernandez-Losa J Aceves Luquero CI de la Cruz-Morcillo MA Ramirez-Castillejo C Callejas-Valera JL Arriaga A Aranburo AF Ramon y Cajal S Silvio Gutkind J Sanchez-Prieto R c-Abl activates p38 MAPK independently of its tyrosine kinase activity: Implications in cisplatin-based therapy Int J Cancer 2008 122 289 297 10.1002/ijc.23063 17893873

[B54] Mahalingam D Keane M Pirianov G Mehmet H Samali A Szegezdi E Differential activation of JNK1 isoforms by TRAIL receptors modulate apoptosis of colon cancer cell lines Br J Cancer 2009 100 1415 1424 10.1038/sj.bjc.6605021 19352384 PMC2694422

[B55] Jin Z El-Deiry WS Distinct signaling pathways in TRAIL- versus tumor necrosis factor-induced apoptosis Mol Cell Biol 2006 26 8136 8148 10.1128/MCB.00257-06 16940186 PMC1636728

[B56] Varfolomeev E Maecker H Sharp D Lawrence D Renz M Vucic D Ashkenazi A Molecular determinants of kinase pathway activation by Apo2 ligand/tumor necrosis factor-related apoptosis-inducing ligand J Biol Chem 2005 280 40599 40608 10.1074/jbc.M509560200 16227629

[B57] Sun BK Kim JH Nguyen HN Oh S Kim SY Choi S Choi HJ Lee YJ Song JJ MEKK1/MEKK4 are responsible for TRAIL-induced JNK/p38 phosphorylation Oncol Rep 2011 25 537 544 21152872 10.3892/or.2010.1079 PMC3041593

